# Comparison of Nursing Home Hearing Handicap Index with Audiological Findings: A Presbycusis Study

**DOI:** 10.1155/2012/423801

**Published:** 2012-10-24

**Authors:** M. H. Nilforoush, A. A. Nasr Esfahani, R. Ishaghi, M. Sepehrnejad

**Affiliations:** ^1^Audiology Department, School of Rehabilitation Sciences, Isfahan University of Medical Sciences, Isfahan 8174673461, Iran; ^2^Audiology Department, School of Rehabilitation Sciences, Tehran University of Medical Sciences, Tehran 141556559, Iran; ^3^Department of Community Medicine, School of Medicine, Isfahan University of Medical Sciences, Isfahan 8174673461, Iran

## Abstract

Hearing evaluation usually includes hearing threshold assessment, middle ear function, and word recognition tests that lead to an accurate result of peripheral and central auditory system. However, they have some limitations because they cannot fully encompass all aspects of hearing loss problems. Using self-assessment approach, via a questionnaire or telephone survey, is one of the easiest methods to study hearing loss in population. In this research, 60 nursing home residents (27 females and 33 males) ranging from 55 to 85 years with a mean age of 71 ± 5.5 were studied via completing self-assessment questionnaire by the elderly cases (NHHI self-version) and the other one was filled by the nursing home personnel (NHHI staff-version). The effects of the hearing loss level on the self- and staff-version scores indicated that there is a significant relationship between self- and staff-version with hearing loss levels (*P* < 0.05) in male and female. Results from this study demonstrate the usefulness of NHHI questionnaire for evaluating hearing handicap of aged people and it may be a useful adjunct in setting up treatment and determining proper care.

## 1. Introduction

Hearing evaluation usually includes hearing threshold assessment, middle ear function, and word recognition tests that lead to an accurate result of peripheral and central auditory system [1–3]. However, they have some limitations because they cannot fully encompass all aspects of hearing loss problems. Communicative ability of patients with sensory-neural hearing loss heavily depends on important factors like acceptance or denial of hearing loss, general communication skills, emotional adaptation, friends and family condition. Because of these nonauditory factors; audiological tests could not evaluate individual ability for communication and participation. Therefore, this group of test in the best conditions can only provide some information of communicative handicap indirectly [4]. Using self-assessment approach, via a questionnaire or telephone survey is one of the easiest methods to study hearing loss in population [5–7]. Many of the self-assessment tools are designed for using in a particular population for instance hearing performance inventory (HPI) questionnaire [8] is appropriate for evaluation of noise induced hearing loss cases, hearing handicap inventory (HHIE) scale [9] is suitable for elderly patients with hearing loss, and communication profile for hearing impaired (CPHI) questionnaire [10] is considered for military studies. These scales are helpful in order to identify the problems and issues related to hearing handicap, understand the communication difficulties, and determine special needs of patients but NHHI focused specifically only on nursing home elderly. In fact, they are not only an objective evaluation method to monitor the progress of rehabilitation process but also provide invaluable information about awareness and consultation for individuals and their families [11]. Nursing home staffs just ask a few questions about hearing problems during completing every case profile and obviously it is not enough to find hearing impaired persons [12–14]. Using hearing aids with environmental modification in nursing home might improve general situation and specially hearing condition for this group of people. Therefore, hearing handicap self-assessment results, audiometric findings and patient's tending to use hearing aids or any assistive listening devices reflect their aural rehabilitation needs more clearly [15]. Nursing home residents problems were focused in NHHI (Nursing Home Hearing Handicap Index) questionnaire which developed by Show and Nerbonne in 1977. The 20 items questionnaire divided into two versions: the self-version which was completed by residents (10 items) and staff-version that should be fulfilled by the nursing home staff (10 items). In addition, all items of two versions are the same [16]. In this research we aimed to compare NHHI questionnaire results with PTA finding of nursing home residents.

## 2. Methods

60 nursing home residents (27 females and 33 males) ranging from 55 to 85 years with a mean age of 71 ± 5.5 were selected. After case history and otoscopy examination, pure tone audiometry test was conducted via Madsen OB822 audiometer to identify hearing loss, and tympanometry test was performed by Interacoustics AT235H tympanometer to rule out any middle ears disorders. Then NHHI questionnaire [16] was completed by the elderly cases (NHHI self-version) and the other one was filled by the nursing home personnel (NHHI staff-version). Kruskal-Wallis statistical test and Pearson's correlation coefficient were used to analyze data. *P* < 0.05 was considered statistically significant.

## 3. Results

Hearing loss level data is found in Table 1 divided into severity of hearing loss and gender.

The analysis of questionnaire results showed that the average score of self-version part was 33.80% ± 27.01 and the average score of staff-version was 34.54% ± 26.48. These results are listed in Table 2.

The effects of the hearing loss level on the self- and staff-version scores indicated that there is a significant relationship between self- and staff-version with hearing loss levels (*P* < 0.05). In other words, the severity of hearing loss becomes greater as the questionnaire score goes up and followed by hearing handicap is also increasing in elderly. This relationship is not significant in male group (*P* > 0.05) however it is significant in the female group (*P* < 0.05) in the self-version. On the other hand, the relationship between the hearing loss levels and staff-version is significant in both men and women groups (*P* < 0.05) as can be found in Table 3.

In testing the relationship between NHHI results and audiological findings by using Chi-square and Pearson's correlation coefficient, the results revealed significantly correlated with each other at a statistically significant level (*P* value) of <0.05 as shown in Table 4.

## 4. Discussion

Outcomes from a hearing handicap scale can give more information about the patient's understanding of hearing loss effects on daily life. If the resident reports a considerable handicap, then auditory rehabilitation will be suggested. Nursing home staffs that are close to resident can also complete a hearing handicap scale. Results can provide essential information on how others distinguish the resident's hearing loss and audiological rehabilitation necessitate. In the present study, we used NHHI questionnaire and compared its results with PTA findings. Moreover, we investigated the correlation of hearing impairment severity on NHHI (two versions) scores in nursing home elderly. Because of significant Pearson's correlation coefficient results (*P* < 0.05) between both versions with hearing loss levels and as other studies suggested [17–19] it can be inferred in cases who were not able to respond scale, staff answers might be considered. In this study results indicated using self-report scale could be valuable in order to prepare rehabilitation needs of elderly before hearing handicap was grown up and its difficulties came out.

## 5. Conclusion

Results from this study demonstrate the usefulness of NHHI questionnaire for evaluating hearing handicap of aged people and it may be a useful adjunct in setting up treatment and determining proper care.

**Limitation of Study**

In spite of searching for new resources in this field and no newer-revised version of the NHHI questionnaire, references might be somewhat old, but nevertheless they are reliable in this area.

## Figures and Tables

**Table 1:** Hearing loss degree (male/female) *n* = 60.

Hearing loss degree	Normal	Mild	Slight	Moderate	Moderately severe	Severe	Profound
Male	9	6	5	7	3	1	2
Female	6	7	4	5	2	2	1

Total (%)	25	21.6	15	20	8.3	5	5

**Table 2:** Mean and SD of NHHI results in both groups (male/female).

Sex	Self-version		Staff-version	
	Mean	SD	Mean	SD
Male	20.36	22.20	26.08	23.26
Female	47.11	28.91	42.43	37.79

Total	33.80%	27.01%	34.54%	29.48%

**Table 3:** Mean values of self-version and staff-version (NHHI) (*n* = 60).

Questionnaire	Hearing loss level							
		Normal	Slight	Mild	Moderate	Moderately severe	Severe	Profound
Self-version	Male	3.50	16.08	28.01	27.15	39.01	40	42
	Female	1.10	12.14	30.44	45.27	75.03	74.29	80

Staff-version	Male	7.03	11.70	40.21	39.18	48.24	43	51
	Female	2.40	1.80	8.07	62.44	71.08	77	82

**Table 4:** Pearson's correlation coefficient results (*r*) in NHHI results with audiological findings.

	Self-version	Staff-version	Hearing loss level
Self-version	—	0.816	0.751
Staff-version	0.816	—	0.762
Hearing loss level	0.751	0.762	—
*P* value	<0.05	<0.05	<0.05
